# Evaluation of Anti-inflammatory Effects of Steroids and Arthritis-Related Biotherapies in an *In Vitro* Coculture Model with Immune Cells and Synoviocytes

**DOI:** 10.3389/fimmu.2016.00509

**Published:** 2016-11-17

**Authors:** Mélissa Noack, Ndiémé Ndongo-Thiam, Pierre Miossec

**Affiliations:** ^1^Immunogenomics and Inflammation Research Unit, EA 4130, Edouard Herriot Hospital, Hospices Civils de Lyon and University Claude Bernard Lyon 1, Lyon, France

**Keywords:** methylprednisolone, biotherapies, rheumatoid arthritis, cell interaction, pro-inflammatory cytokines

## Abstract

**Background:**

During rheumatoid arthritis (RA), steroids and biotherapies are used alone and combined. Efficacy has been established in clinical trials but their differential effects at the cellular level are less documented. The aim was to study these cellular effects using an *in vitro* model with synoviocytes interacting with peripheral blood mononuclear cells (PBMC) to reproduce the interactions in the RA synovium.

**Methods:**

Activated-PBMC were cocultured with RA synoviocytes during 48 h. A dose–response of methylprednisolone (MP) was tested and different biotherapies (Infliximab, Etanercept, Adalimumab, Tocilizumab, Abatacept, and Rituximab) were added alone or in combination with MP. Cytokine production (IL-17, IL-6, IL-1β, IFN-γ and IL-10) was measured by ELISA.

**Results:**

Addition of MP to cocultures inhibited the production of all cytokines. The response to the biotherapies alone was treatment-dependent. IL-17 production was inhibited only by Tocilizumab (*p* = 0.004), while IL-6 was decreased only by Infliximab (*p* ≤ 0.002). IL-1β level was affected in all conditions (*p* ≤ 0.03). IFN-γ production was mainly decreased by Infliximab (*p* = 0.004) and IL-10 by Infliximab and Tocilizumab (*p* ≤ 0.004). The combination MP and biotherapies did not induce an additional effect on pro-inflammatory cytokine inhibition. The combination MP and biotherapies induced a higher IL-10 secretion than MP alone, mainly with Rituximab.

**Conclusion:**

Steroids inhibited the secretion of all cytokines, and low doses were as potent. The anti-inflammatory effect of biotherapies was dependent on their mechanism of action. MP and biotherapy combination did not enhance the inhibitory effect on pro-inflammatory cytokines but could have a beneficial effect by increasing IL-10 production.

## Introduction

Rheumatoid arthritis (RA) is a chronic inflammatory disease leading to joint destruction (1). Even if its etiology is not yet clarified, different treatments are available to treat the clinical symptoms. Steroids (also named glucocorticoids or corticosteroids) are the oldest and the most classical anti-inflammatory therapy used for many chronic inflammatory diseases other than RA. They act by inhibiting multiple inflammatory genes (encoding cytokines, chemokines, etc.) that are activated during chronic inflammation (2–4). However, their use is associated with adverse events mainly infections specifically at high dose. Moreover, non-responsiveness or resistance can be observed (5). Another key drug is methotrexate (MTX), the most common treatment of RA. MTX improves clinical parameters in RA patients but severe adverse events could lead to discontinuation (6–8).

The heterogeneity of the response to steroids and MTX has led to a combination therapy with biotherapies. In RA, anti-TNF inhibitors are the first and most used biotherapy. Many studies have described the improvement of symptoms and physical functions (9). Nevertheless, adverse events, mainly elevated risk of infections (10), are known and prevention measures are in place. Current studies focus on the clinical level of treatment efficacy, but the mode of action at the cellular level is less documented.

In the RA joint synovium, the inflammation leads to the recruitment of immune cells that interact with local mesenchymal cells as synoviocytes. These interactions contribute to the chronicity of inflammation, notably by increasing the pro-inflammatory cytokine production and the cell survival of synoviocytes (11–13). In our previous studies, the effects of cell interactions on pro-inflammatory cytokine production were studied using an *in vitro* model of coculture between mesenchymal cells and peripheral blood mononuclear cells (PBMC). These studies showed that cell interactions were critical for massive pro-inflammatory cytokine secretion. The use of an autologous system validated this model as mimicking the *in vivo* situation (14, 15).

Herein, in the RA inflammatory context, the aim was to compare the effects of all the current biotherapies against TNF, IL-6, CD20, and CTLA4, with or without steroids by using this *in vitro* cell–cell interaction model looking at cytokine production.

## Materials and Methods

### Samples

Rheumatoid arthritis synoviocytes were obtained from synovial tissue of RA patients undergoing joint surgery and who fulfilled the American College of Rheumatology criteria for RA (16). Synovial tissue was minced into small pieces and then adhered in 6-well plates in Dulbecco’s modified Eagle’s medium (DMEM; Eurobio, Courtaboeuf, France), supplemented with 10% fetal bovine serum (FBS; Life Technologies, Carlsbad, USA), 2mM l-glutamine, and 100 U/ml penicillin/streptomycin. Cells were maintained at 37°C in a humidified 5% carbon dioxide incubator and used between passages 4 to 9. PBMC from healthy donors were isolated by Ficoll-Hypaque (Eurobio, Courtaboeuf, France) density-gradient centrifugation. Each individual signed an informed consent form. The protocol was approved by the Ethics Committee of the Hospitals of Lyon for the protection of persons participating in biomedical research under number AC-2010-11-64.

### Coculture Assays

Coculture was initiated by seeding RA synoviocytes overnight in 96-well plates at a density of 2 × 10^4^ cells/well in RPMI 1640 medium (Eurobio, Courtaboeuf, France) supplemented with 10% human AB serum, 2mM l-glutamine, and 100 U/ml penicillin/streptomycin (complete RPMI). The next day, healthy PBMC (1 × 10^5^ cells/well) were pre-incubated in complete RPMI with or without different treatments and then seeded corresponding to 5:1 ratio, in the presence of phytohemagglutinin (PHA, 5 μg/ml). After 48 h, supernatants and PBMC were collected for analysis (17).

### Treatments

A dose–response of Methylprednisolone (MP) was done with 0, 0.001, 0.01, 0.1, 1, and 10 μg/ml. The concentration of 0.01 μg/ml was used in combination with biotherapies. Two concentrations of biotherapies were used in coculture, 10 and 100 μg/ml. Infliximab (Remicade, anti-TNF, Merk), Tocilizumab (Roactemra, anti-IL-6 receptor, Roche-Chugai), Abatacept (Orencia, CTLA4 Ig, BMS), Rituximab (Mabthera, anti-CD20, Roche), Etanercept (Enbrel, anti-TNF, Amgen), and Adalimumab (Humira, anti-TNF, Abbvie) were tested.

### Enzyme-Linked Immunosorbent Assays

IL-17A, IL-6, IL-1β, IFN-γ, and IL-10 production was evaluated from culture supernatants with commercially available Duoset enzyme-linked immunosorbent assays (ELISA) kits, according to the manufacturer’s instructions (R&D system, Minneapolis, MN, USA).

### Statistical Analysis

Statistical analyses were performed using paired Wilcoxon test. All analyses were performed with Graph Pad Prism 6 software. *p* values less than or equal to 0.05 were considered as significant.

## Results

### Principle of the *In Vitro* Model

**Figure 1:**
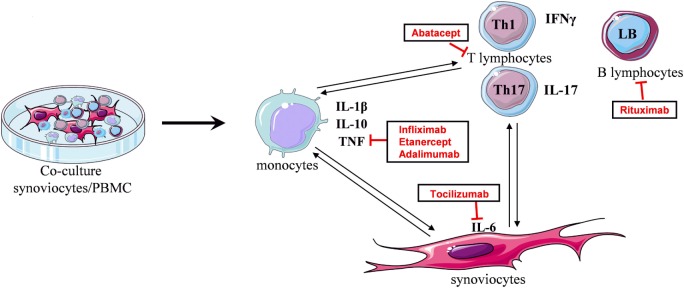
**Principle of the *in vitro* coculture model**. Coculture between synoviocytes and activated-PBMC leads to cell interactions promoting the secretion of cytokines. Cell–cell contact induces IL-1β, TNF, or IL-10 production, mainly by monocytes. In turn, TNF and IL-1β stimulate synoviocytes to produce IL-6. Secreted cytokines influence T lymphocyte differentiation, as IL-6 and IL-1β for Th17 cells, and then interactions with synoviocytes promote the release of their cytokine, as IL-17 which, in turn, stimulates synoviocytes. The coculture reproduces the cell interactions existing in the inflammatory site and the cytokine environment. Using this model, the effect of treatments on cytokine production resulting from cell interactions can be studied.

During chronic inflammatory arthritis, immune cells infiltrate the inflammatory synovium and interact with local mesenchymal cells, as synoviocytes. This promotes the secretion of various cytokines and chemokines. To mimic this event, an *in vitro* model of coculture was used. In the RA context, synoviocytes from patients were cultured in the presence of activated-PBMC. These cell–cell interactions lead to massive cytokine production, compared to PBMC alone. In control condition, the cell contact between synoviocytes and healthy resting-PBMC is sufficient to induce IL-6 and IL-1β production. Nevertheless, a high IL-17 secretion is only obtained in coculture with activated-PBMC. This model was validated by using an autologous system, that is PBMC and synoviocytes from the same RA patients (14). Using this model, the effect of treatments on cytokine production resulting from cell interactions could be studied (Figure 1).

### Effects of Methylprednisolone

**Figure 2:**
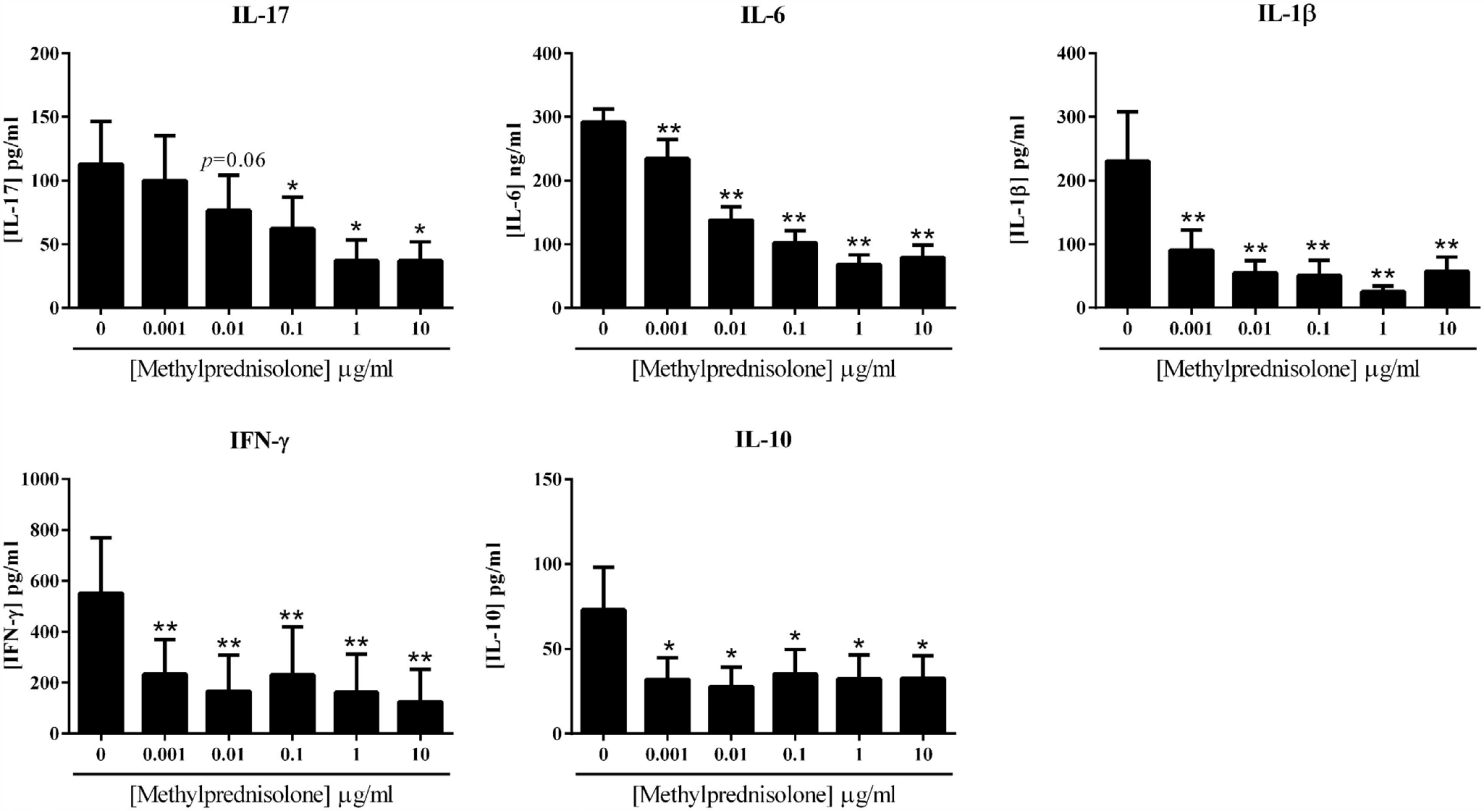
**Effect of the dose–response of methylprednisolone on cytokine production**. Healthy PBMC activated by PHA (5 μg/ml) were pre-incubated or not with different doses of methylprednisolone. Then, PBMC were cocultured with RA synoviocytes at a ratio 5:1 for 48 h. The production of IL-17, IL-6, IL-1β, IFN-γ, and IL-10 in cell supernatants was measured by enzyme-linked immunosorbent assay (ELISA). *p ≤ 0.05. Results are represented as mean ± SEM, *n* = 8 experiments from six different RA patients.

The effect of MP was evaluated in the coculture system, using a dose–response curve. Different concentrations of MP were tested: 0, 0.001, 0.01, 0.1, 1, and 10 μg/ml. The impact on pro- and anti-inflammatory cytokine production was measured in the supernatants of cultures after 48 h. As observed in Figure 2, IL-17 secretion was significantly decreased from 0.1 μg/ml (62.3 ± 24.7 vs. 112.8 ± 33.8 pg/ml, *p* = 0.03), but there was already a decreased production starting from 0.01 μg/ml (76.6 ± 27.8 vs. 112.8 ± 33.8 pg/ml, *p* = 0.06). The inhibition followed a dose–response. IL-6, IL-1β, and IFN-γ production significantly decreased starting with the lowest concentration of 0.001 μg/ml (234.7 ± 30.2 vs. 291.8 ± 20.2 ng/ml, for IL-6; 90.4 ± 31.7 vs. 230.5 ± 77.8 pg/ml, for IL-1β, *p* = 0.008; 234.4 ± 136.5 vs. 552.3 ± 217.6 pg/ml, for IFN-γ, *p* = 0.03). The dose effect was clear for IL-6 secretion and almost for IL-1β; nevertheless, for IFN-γ, the maximum effect was reached from the lowest concentration.

If MP inhibited well the production of pro-inflammatory cytokines, the secretion of the anti-inflammatory cytokine IL-10 was also inhibited by the addition of MP. This decreased production was significant from the lowest concentration of 0.001 μg/ml (32.0 ± 12.8 vs. 70.8 ± 23.6 pg/ml, *p* = 0.02) and was not clearly dose-dependent.

These results confirmed the anti-inflammatory potential of MP in *in vitro* system, but showed that MP also decreased the production of IL-10.

### Effects of Biotherapies Alone

**Figure 3:**
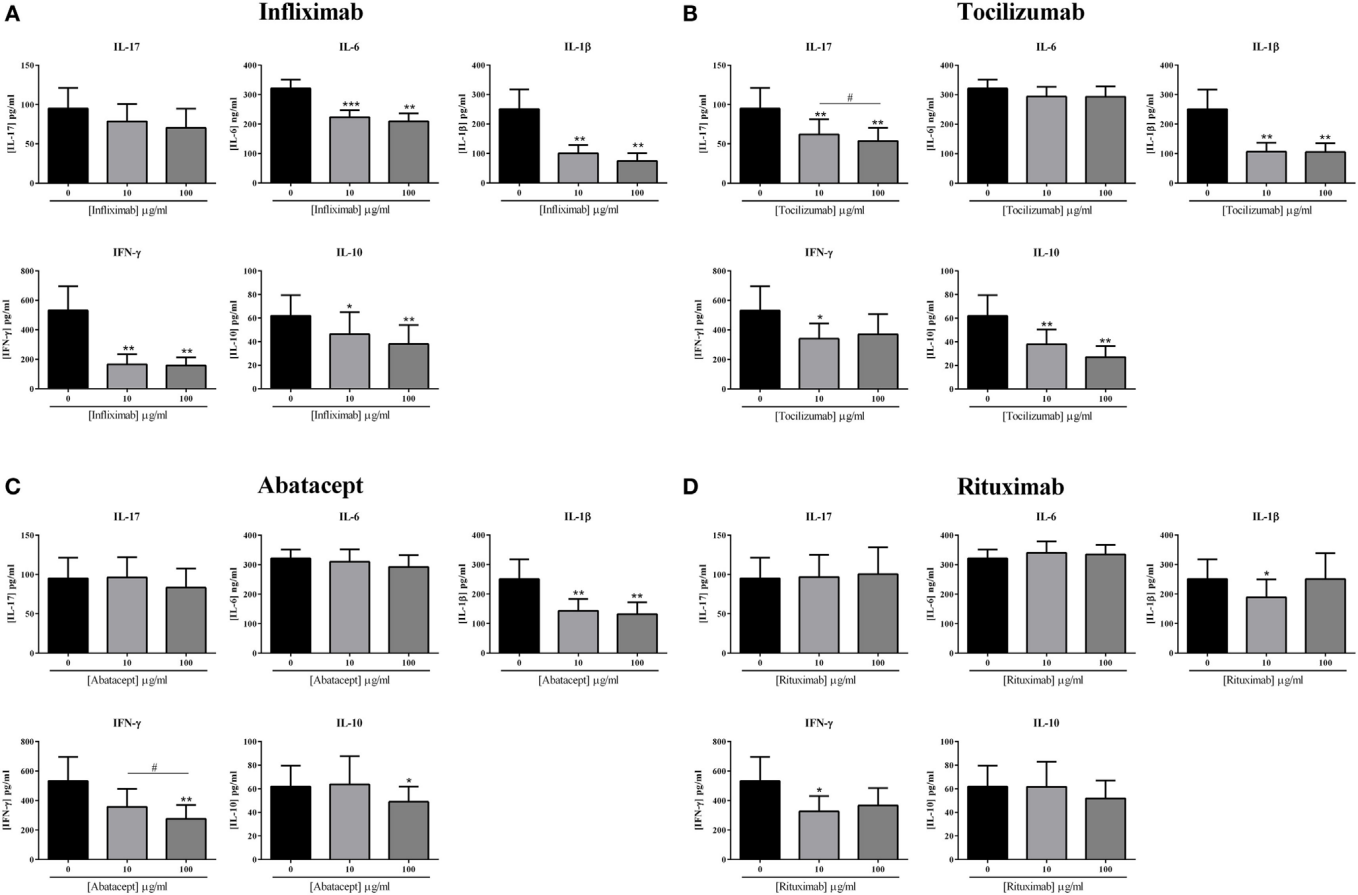
**Effect of biotherapies on cytokine production**. Healthy activated-PBMC were pre-incubated or not with two doses of biotherapies, 10 or 100 μg/ml and cocultured with RA synoviocytes at a ratio 5:1 for 48 h. Infliximab **(A)**, Tocilizumab **(B)**, Abatacept **(C)**, and Rituximab **(D)** were tested. After 48 h, the production of IL-17, IL-6, IL-1β, IFN-γ, and IL-10 in cell supernatants was measured by ELISA. *p ≤ 0.05. Results are represented as mean ± SEM, *n* = 11 experiments from six different RA patients.

The effects of the current biotherapies for RA treatment were first tested alone. After preliminary experiments, two doses, 10 and 100 μg/ml, were selected and tested for their effect on cytokine production. As shown in Figure 3, the effects of the addition of biotherapies in coculture were measured on the production of the pro-inflammatory cytokines IL-17, IL-6, IL-1β, and IFN-γ and the anti-inflammatory cytokine IL-10.

Infliximab is a monoclonal antibody that binds TNF. Infliximab decreased the production of IL-17 at 10 μg/ml, without reaching significance (78.5 ± 22.1 vs. 94.8 ± 26.1 pg/ml, *p* = 0.07, Figure 3A) and also at 100 μg/ml (70.3 ± 24.3 vs. 94.8 ± 26.1 pg/ml, *p* = 0.055, Figure 3A). On the other hand, IL-6 production was decreased by around 30–35%, without a clear dose–response (223.7 ± 23.1 vs. 321.1 ± 30.1 ng/ml, for 10 μg/ml, *p* = 0.001; 209.2 ± 27.5 vs. 321.1 ± 30.1 ng/ml, for 100 μg/ml, *p* = 0.002, Figure 3A). The secretion of IL-1β and IFN-γ was the most inhibited by the addition of Infliximab, with a decrease around 60–70% (100.8 ± 28.1 vs. 250.7 ± 66.7 pg/ml, for 10 μg/ml and 74.5 ± 26.8 vs. 250.7 ± 66.7 pg/ml, for 100 μg/ml, for IL-1β, *p* = 0.002; 166.0 ± 67.9 vs. 532.0 ± 163.9 pg/ml, for 10 μg/ml and 158.0 ± 55.9 vs. 532.0 ± 163.9 pg/ml, for 100 μg/ml, for IFN-γ, *p* = 0.004, Figure 3A). Furthermore, Infliximab also decreased by about 30% the production of IL-10 (46.3 ± 18.7 vs. 62.8 ± 17.5 pg/ml, for 10 μg/ml, *p* = 0.05; 38.0 ± 16.2 vs. 62.8 ± 17.5 pg/ml, for 100 μg/ml, *p* = 0.002, Figure 3A).

Tocilizumab is a monoclonal antibody that binds the IL-6 receptor. Tocilizumab inhibited significantly the production of IL-17 either at 10 μg/ml (62.0 ± 19.4 vs. 94.8 ± 26.1 pg/ml, *p* = 0.004, Figure 3B) or at 100 μg/ml (53.7 ± 16.7 vs. 94.8 ± 26.1 pg/ml, *p* = 0.004, Figure 3B), with a dose effect (*p* = 0.04, Figure 3B). While IL-1β production was also significantly decreased by the addition of Tocilizumab (106.5 ± 30.8 vs. 250.7 ± 66.7 pg/ml, for 10 μg/ml; 105.1 ± 30.4 vs. 250.7 ± 66.7 pg/ml, for 100 μg/ml, *p* = 0.002, Figure 3B), without a clear dose effect, the secretion of IL-6 was similar to the control (293.4 ± 33.1 vs. 321.1 ± 30.1 ng/ml for 10 μg/ml; 293.3 ± 34.9 vs. 321.1 ± 30.1 ng/ml, for 100 μg/ml, Figure 3B). The IFN-γ secretion was significantly reduced with 10 μg/ml (340.1 ± 105.1 vs. 532.0 ± 163.9 pg/ml, *p* = 0.03, Figure 3B) but without reaching significance with 100 μg/ml (370.2 ± 137.0 vs. 532.0 ± 163.9 pg/ml, *p* = 0.09, Figure 3B), suggesting that high dose is not necessarily more efficient. The production of IL-10 was also significantly decreased by the addition of Tocilizumab (37.9 ± 12.6 vs. 62.8 ± 17.5 pg/ml, for 10 μg/ml; 27.1 ± 9.2 vs. 62.8 ± 17.5 pg/ml, for 100 μg/ml, *p* = 0.002, Figure 3B).

Two biotherapies targeting specifically cells were also tested, Abatacept and Rituximab. Abatacept is a fusion protein (CTLA-4-Ig) which interacts with B7 (ligand of CD28 which is a T cell activation molecule). The addition of Abatacept did not affect IL-17 and IL-6 secretion at 10 μg/ml (96.1 ± 25.8 vs. 94.8 ± 26.1 pg/ml for IL-17; 309.5 ± 42.1 vs. 321.1 ± 30.1 ng/ml for IL-6, Figure 3C) and at 100 μg/ml (83.2 ± 24.5 vs. 94.8 ± 26.1 pg/ml for IL-17; 291.9 ± 40.6 vs. 321.1 ± 30.1 ng/ml for IL-6, Figure 3C). The production of IL-1β was significantly decreased by about 45%, without a dose–effect (143.1 ± 40.4 vs. 250.7 ± 66.7 pg/ml, for 10 μg/ml; 132.0 ± 40.5 vs. 250.7 ± 66.7 pg/ml, for 100 μg/ml, *p* = 0.002, Figure 3C). IFN-γ secretion was altered in a dose-dependent pattern, as there was a significant decrease between 10 and 100 μg/ml (356.6 ± 123.4 vs. 274.7 ± 95.2 pg/ml, respectively, *p* = 0.04, vs. 532.0 ± 163.9 pg/ml for the control, Figure 3C). IL-10 was decreased only with the higher dose of 100 μg/ml (48.9 ± 13.1 vs. 62.8 ± 17.5 pg/ml, *p* = 0.03, Figure 3C).

Rituximab is a monoclonal antibody against CD20. As observed in Figure 3D, Rituximab had no effect on the secretion of IL-17, IL-6, and IL-10. The concentration of 10 μg/ml decreased significantly IL-1β production (188.8 ± 60.6 vs. 250.7 ± 66.7 pg/ml, *p* = 0.04) and also IFN-γ secretion (370.2 ± 137.0 vs. 532.0 ± 163.9 pg/ml, *p* = 0.02). At 100 μg/ml, their production was not significantly decreased and as for Tocilizumab, this suggests that increasing the dose may not be necessary and even could have an opposite effect.

In summary, a global inhibitory effect of all biotherapies was observed but with differences between treatments. Infliximab and Tocilizumab had a higher and broader effect than Abatacept and Rituximab. This could be explained by the broader effect of Infliximab and Tocilizumab acting on cytokines and their receptors contrasting with the cell specificity of Abatacept and Rituximab.

### Effects of Various Anti-TNF Comparisons

**Figure 4:**
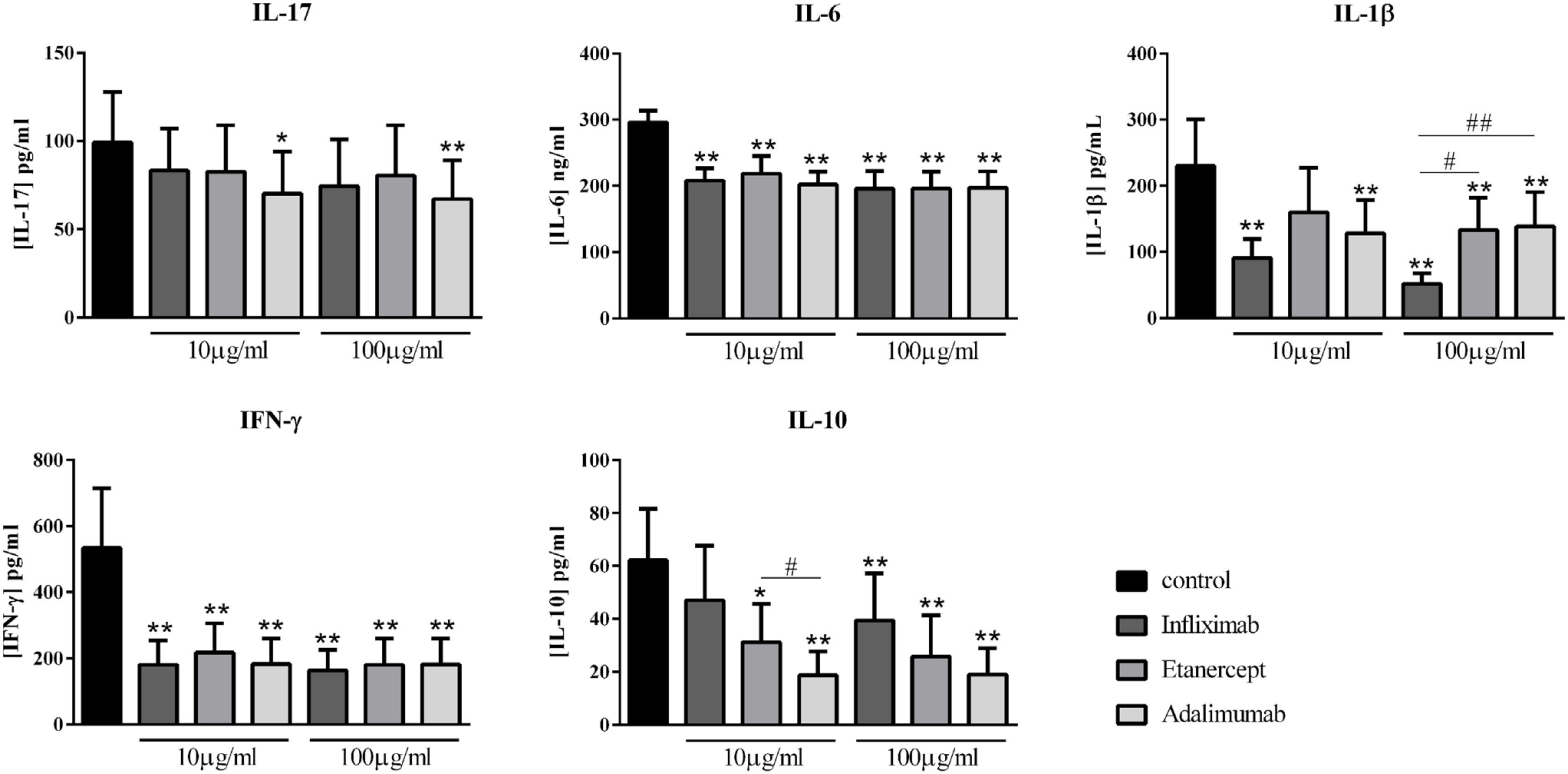
**Comparison of different anti-TNF biotherapies**. Healthy activated-PBMC were pre-incubated or not with two doses of anti-TNF biotherapies, 10 μg/ml or 100 μg/ml and cocultured with RA synoviocytes at a ratio 5:1 for 48 h. Infliximab, Etanercept, and Adalimumab were compared. After 48 h, the production of IL-17, IL-6, IL-1β, IFN-γ, and IL-10 in cell supernatants was measured by ELISA. *^,#^*p* ≤ 0.05. *Compares anti-TNF vs. control and ^#^compares the different anti-TNF. Results are represented as mean ± SEM, *n* = 10 experiments from six different RA patients.

Various inhibitors of TNF are currently used with different modes of action. Infliximab was compared to two other TNF inhibitors, Etanercept, a soluble receptor, and Adalimumab, a monoclonal antibody like Infliximab. As observed in Figure 4, at both concentrations, only Adalimumab decreased significantly the secretion of IL-17 by about 30% (70.1 ± 24.0 vs. 99.2 ± 28.4 pg/ml for 10 μg/ml; and 67.1 ± 22.0 vs. 99.2 ± 28.4 pg/ml for 100 μg/ml, *p* = 0.008). The three biotherapies had a similar effect on IL-6 and IFN-γ, with an inhibition by about 30 and 70%, respectively, with no clear dose effect (Figure 4). For IL-1β, Infliximab was the most potent at both concentrations of the three inhibitors (51.8 ± 15.8 vs. 230.3 ± 70.3 pg/ml for Infliximab, *p* = 0.004; 133.0 ± 49.1 vs. 230.3 ± 70.3 pg/ml for Etanercept, *p* = 0.004; and 138.4 ± 52.0 vs. 230.3 ± 70.3 pg/ml for Adalimumab, *p* = 0.004). The three anti-TNF inhibited significantly the IL-10 production, without a dose–effect. A difference between the three biotherapies was observed, significantly only at 10 μg/ml. Infliximab decreased less IL-10 production (47.0 ± 20.7 vs. 63.1 ± 19.3 pg/ml for 10 μg/ml, *p* = 0.07; and 39.4 ± 17.9 vs. 63.1 ± 19.3 pg/ml for 100 μg/ml, *p* = 0.004), followed by Etanercept (31.2 ± 14.5 vs. 63.1 ± 19.3 pg/ml for 10 μg/ml, *p* = 0.03; and 25.8 ± 15.5 vs. 63.1 ± 19.3 pg/ml for 100 μg/ml, *p* = 0.008); and the most inhibitory effect was obtained with Adalimumab (18.7 ± 9.0 vs. 63.1 ± 19.3 pg/ml for 10 μg/ml, *p* = 0.004; and 18.9 ± 9.9 vs. 63.1 ± 19.3 pg/ml for 100 μg/ml, *p* = 0.004). Overall, the three anti-TNF displayed a similar effect. Nevertheless, Infliximab was the more potent on IL-1β inhibition and less than the others on IL-10 secretion.

### Combination of MP and Biotherapies

**Figure 5:**
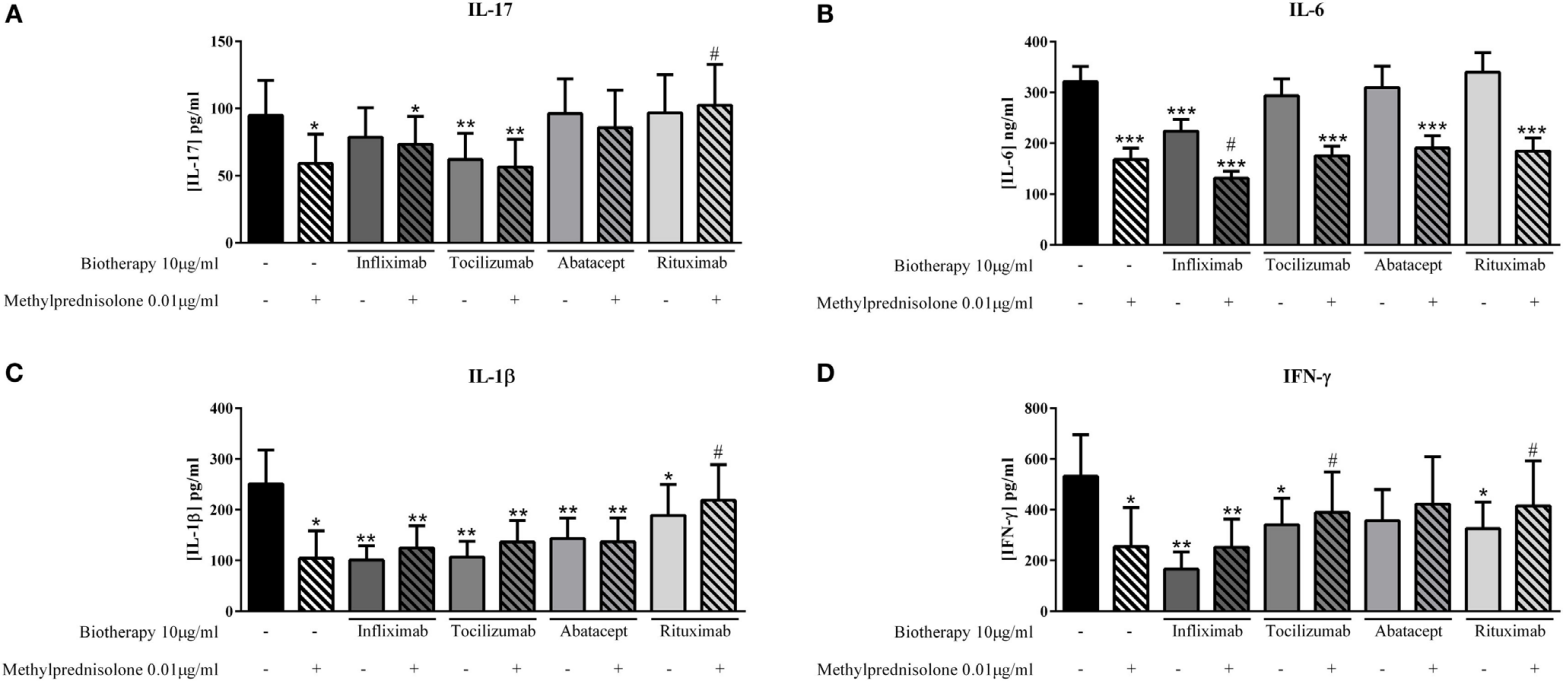
**Effect of the combination of methylprednisolone and biotherapy on pro-inflammatory cytokine production**. Healthy activated-PBMC were pre-incubated or not with methylprednisolone alone (0.01 μg/ml), biotherapy alone (10 μg/ml), or with the combination of both treatments. Then, PBMC were cocultured with RA synoviocytes at a ratio 5:1 for 48 h. Infliximab **(A)**, Tocilizumab **(B)**, Abatacept, **(C)** and Rituximab **(D)** were tested. After 48 h, the production of IL-17, IL-6, IL-1β, and IFN-γ in cell supernatants was measured by ELISA. *^,#^*p* ≤ 0.05. *Compares with control and ^#^compares combination MP/biotherapy vs. MP alone. Results are represented as mean ± SEM, *n* = 11 experiments from six different RA patients.

The effect of treatment combination is not well understood in patients, and thus the basis for combining drugs remains unclear. The effect of MP and biotherapies, alone or in combination, was compared. The lowest effective doses of MP, 0.01 μg/ml and of biotherapies, 10 μg/ml, were used alone or in combination to evaluate their impact on cytokine production. MP alone inhibited significantly IL-17 production (58.9 ± 23.4 vs. 94.8 ± 26.1 pg/ml, *p* = 0.02, Figure 5A) as Tocilizumab alone (62.0 ± 19.4 vs. 94.8 ± 26.1 pg/ml, *p* = 0.004, Figure 5A). Infliximab, Abatacept, and Rituximab alone did not inhibit IL-17 production (Figure 5A). In combination, the inhibitory effect of MP and Tocilizumab was not increased and the inhibition was still around 40% (56.5 ± 20.6 vs. 94.8 ± 26.1 pg/ml, *p* = 0.004, Figure 5A). The combination of MP and Infliximab or Abatacept tended to increase IL-17 production compared to MP alone (73.3 ± 20.8 and 85.7 ± 27.8 pg/ml, respectively, vs. 58.9 ± 23.4 pg/ml, *p* = 0.07, Figure 5A). The combination of MP and Rituximab significantly inhibited the MP effect and the level of IL-17 was similar to the control (102.3 ± 30.6 vs. 94.8 ± 26.1 pg/ml, *p* = 0.03, Figure 5A).

For IL-6 secretion, MP and Infliximab alone decreased significantly IL-6 secretion compared to control (167.7 ± 21.0 pg/ml and 78.5 ± 22.1 pg/ml, respectively, vs. 321.1 ± 30.1 pg/ml, *p* = 0.001, Figure 5B); and the combination of both increased significantly the inhibitory effect compared to MP alone (131.3 ± 13.6 vs. 167.7 ± 21.0 pg/ml, *p* = 0.04, Figure 5B). The combination of MP with other biotherapies (Tocilizumab, Abatacept, and Rituximab) did not change the decreased IL-6 production induced by MP alone (Figure 5B).

IL-1β production was decreased in a similar way between MP alone, Infliximab, Tocilizumab, or Abatacept alone and the combination of both (Figure 5C). Nevertheless, Rituximab alone inhibited IL-1β secretion but to a less extent that MP alone, compared to control (188.8 ± 60.6 pg/ml, *p* = 0.04, and 104.5 ± 59.2 pg/ml, *p* = 0.002, respectively, vs. 250.7 ± 77.8 pg/ml); and the combination of both canceled the large inhibitory effect of MP alone (218.4 ± 70.4 vs. 104.5 ± 59.2 pg/ml, *p* = 0.03, Figure 5C).

Except Abatacept, biotherapies (Infliximab, Tocilizumab and Rituximab) or MP alone decreased significantly the production of IFN-γ compared to control (Figure 5D). The combination of MP and Infliximab led to a similar inhibition than MP alone (252.2 ± 110.6 vs. 254.2 ± 169.2 pg/ml compared to 532.0 ± 163.9 pg/ml for control, Figure 5D). The combination of MP with Tocilizumab and Rituximab significantly reduced the inhibition of IFN-γ secretion compared to MP alone (389.3 ± 159.4 pg/ml, *p* = 0.03, and 415.1 ± 178.1 pg/ml, *p* = 0.01, respectively, vs. 254.2 ± 169.2 pg/ml, Figure 5D), and it was the same tendency for the combination of MP and Abatacept (*p* = 0.08, Figure 5D).

Herein, the results showed that the combination even at low doses did not necessarily result in an additional effect on the inhibition of pro-inflammatory cytokine production.

### Effect on IL-10 Secretion

**Figure 6:**
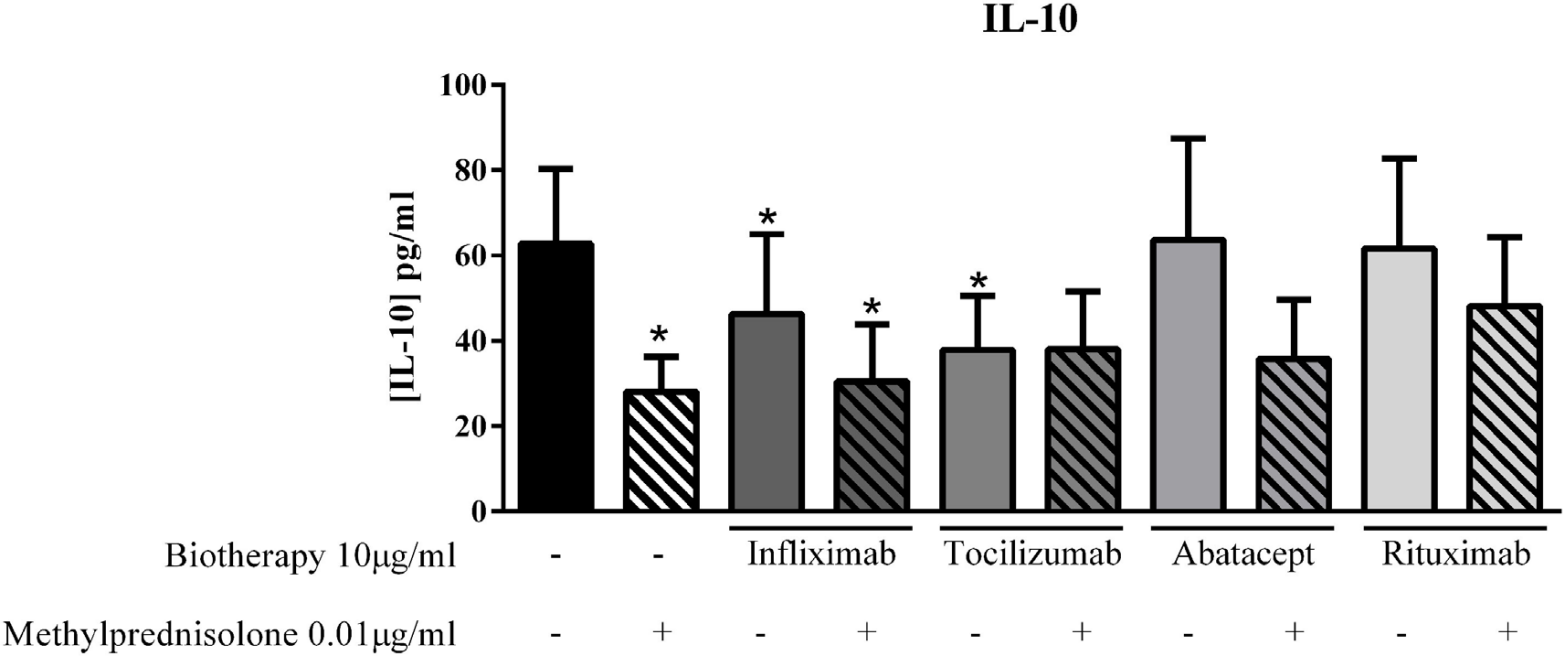
**Effect of the combination of methylprednisolone and biotherapy on IL-10 production**. Healthy activated-PBMC were pre-incubated or not with methylprednisolone alone (0.01 μg/ml), biotherapy alone (10 μg/ml), or with the combination of both treatments. Then, PBMC were cocultured with RA synoviocytes at a ratio of 5:1 for 48 h. Infliximab, Tocilizumab, Abatacept, and Rituximab were tested. After 48 h, the production of IL-10 in cell supernatants was measured by ELISA. *p ≤ 0.05. *Compares with control. Results are represented as mean ± SEM, *n* = 10 experiments from six different RA patients.

Given some unexpected results on pro-inflammatory cytokines with the combination of MP and biotherapy, the next step was to detect a potential effect on the anti-inflammatory cytokine IL-10. The dose of 0.01 μg/ml of MP alone decreased IL-10 production compared to control (28.0 ± 9.1 vs. 62.8 ± 17.5 pg/ml, *p* = 0.01, Figure 6). Infliximab and Tocilizumab alone inhibited significantly the IL-10 secretion (46.3 ± 18.7 pg/ml, *p* = 0.05, and 37.9 ± 12.6 pg/ml, *p* = 0.002, respectively, vs. 62.8 ± 17.5 pg/ml, Figure 6), while Abatacept and Rituximab did not affect the IL-10 level compared to control (63.8 ± 23.7 pg/ml and 61.6 ± 21.2 pg/ml, respectively, vs. 62.8 ± 17.5 pg/ml, Figure 6). In the presence of the combination of MP and biotherapy, the IL-10 level was lower than control but higher than MP alone, mainly with Rituximab, without reaching significance (48.1 ± 16.3 vs. 28.0 ± 9.1 pg/ml, *p* = 0.3, Figure 6). These results indicated that the beneficial effect of combination of MP and biotherapy could come from a reduction of the inhibitory effect of MP on IL-10 production.

## Discussion

In this study, the interest was focused on the effects of steroids and biotherapies alone and combined on cytokine production resulting from cell interactions between mesenchymal cells and immune cells.

Steroids are very effective anti-inflammatory drugs but the adverse effects limit their use. There is no doubt that steroids present a strong potential for frequent and serious side effects, increasing with longer use and higher dose (18). Side effects of chronic steroid therapy are dose-related. At a low-dose (<7.5 mg/day), many studies suggest that steroid treatment is relatively safe in RA (19, 20), and even a very low dose (<5 mg/ml), can be sufficient to maintain remission without severe adverse events (21) but a better clinical response (22–25). To identify their effects at the cellular level, an *in vitro* model based on interactions between mesenchymal cells and PBMC was used to mimic the *in vivo* inflammatory state. A dose–response with MP was done to evaluate its effects on cytokine production. The production of pro-inflammatory cytokines was clearly inhibited by the addition of MP in coculture with differences between cytokines. If IL-17 and IL-6 production decreased according to a dose–response, IL-1β and IFN-γ secretion was already inhibited with the lowest concentration of 0.001 μg/ml. A low dose of MP appears sufficient to induce an inhibitory effect on pro-inflammatory cytokine production. Furthermore, from 10 μg/ml the cytokine production, notably IL-6 and IL-1β, appeared to increase compared to 1 μg/ml. In the clinic, this would suggest high dosage of MP might not be needed to obtain its anti-inflammatory effect through cytokine inhibition and that high doses could have the opposite effect. In addition, the presence of MP even at the lowest dose also inhibited the secretion of IL-10. This may explain the increased risk of infection for the treated patients.

The use of biotherapies during chronic inflammatory diseases, such as RA, has been a major step in improved care. The benefits on clinical symptoms are well established, while the effect at cellular levels is less documented. Our *in vitro* system demonstrated in previous studies that cell interactions between mesenchymal cells including synoviocytes from RA patients or skin fibroblasts from psoriatic patients and immune cells resulted in massive pro-inflammatory cytokine production as observed in the *in vivo* situation (14, 15). Our system was used to evaluate the effect of current biotherapies at the cytokine production level.

TNF is a pro-inflammatory cytokine clearly involved in the pathogenesis of RA (1, 26), notably by stimulating synoviocytes to produce IL-6. As expected, TNF inhibition led to a decreased production of IL-6 which is also a pro-inflammatory cytokine involved in the activation of T cell differentiation. The decrease of IL-6 level could result in the decreased IL-1β and IFN-γ levels. The most used anti-TNF treatments are Infliximab, Etanercept, and Adalimumab, and some studies showed differences in efficacy and adherence between them (27, 28). When compared at the cellular level, the three therapies displayed a rather similar inhibitory effect on pro-inflammatory cytokine production. The major difference appeared on IL-10 production, with Infliximab inhibiting less IL-10 production compared to the two others.

Two-thirds of the RA patients respond to anti-TNF leaving space for other treatment options (27). Tocilizumab is a recombinant humanized monoclonal antibody against the IL-6 receptor (IL-6R), preventing IL-6 binding to both membranous and soluble IL-6R (29, 30). In our *in vitro* system, Tocilizumab decreased significantly IL-1β and IL-17 secretion (26, 31, 32). Both IL-6 and IL-1β are involved in the Th17 differentiation. Thus, the inhibition of IL-6R followed by the decrease of IL-1β could explain the observed inhibition of IL-17 secretion. The secretion of IL-6 was not decreased by the addition of Tocilizumab but such detection is complex. Indeed, in RA patients, the serum level of IL-6 increases after Tocilizumab administration, as IL-6 does not bind its receptor (33). Nevertheless, its signaling pathway is well inhibited as confirmed by the decreased production of CRP in patients.

In addition to cytokine inhibition, other biotherapies target cells. In this way, two other biotherapies, Abatacept and Rituximab were tested on cytokine production. Abatacept is a fusion protein of the extracellular domain of cytotoxic T-lymphocyte-associated protein 4 (CTLA-4) and the Fc region of IgG1. It prevents T cell activation by CD28 by increasing the inhibitory effect of CTLA-4 on T cell activation (34). In our *in vitro* system, the major inhibited cytokine was IL-1β. The decrease of IFN-γ production could be explained by the direct effect of Abatacept on T cell activation. In addition, IL-1β is involved in IFN-γ secretion by T cells (35, 36) and NK cells (37). Thus, Abatacept may have a direct effect on T cell activation and an indirect effect by inhibiting IL-1β production.

Rituximab is directed against CD20 and induces the death of B cells (38). Herein, Rituximab decreased only IL-1β and IFN-γ production, at low dose. As Rituximab acts directly on B cells, it could be interesting to study the *in vitro* effect on immunoglobulin production. Indeed, patients positive for anti-citrullinated protein antibodies (ACPA) appear to respond better to Rituximab (39–41).

Steroids and biotherapies are two different ways to diminish inflammation, by reducing cytokine production. The first conclusion is that high doses were not more efficient than low doses, for both steroids and biotherapies. The effect of their combination each at low dose is more complex. Addition of a biotherapy seemed to reduce the effect of MP alone. At the same time, the combination reduced the inhibitory effect on IL-10 production of the two treatments used alone. At the end, the combination may increase the net anti-inflammatory effects of IL-10 with a suppressor effect on numerous pro-inflammatory cytokines, such as TNF, IL-1β, or IL-6, all involved in RA pathogenesis. The next step of these studies will be to evaluate this time the effects of MTX alone and combined with biotherapies, as done routinely in patients with RA and other inflammatory diseases.

## Author Contributions

MN carried out the experiments and drafted the manuscript. NN-T participated in the experiments. PM conceived the study and reviewed the manuscript. All the authors read and approved the final manuscript.

## Conflict of Interest Statement

The authors declare that the research was conducted in the absence of any commercial or financial relationships that could be construed as a potential conflict of interest.
